# Rapid Detection of N9 Subtype Avian Influenza Viruses Using an NA-Targeting Lateral Flow Immunoassay

**DOI:** 10.3390/v18080879

**Published:** 2026-08-11

**Authors:** Qingmei Li, Yaning Sun, Yuhang Zhang, Zekun Meng, Yunrui Xing, Lu Fan, Jifei Yang, Junqing Guo

**Affiliations:** 1 Institute for Animal Health, Henan Academy of Agricultural Sciences, Zhengzhou 450002, China; liqingmei@hnagri.org.cn (Q.L.); happylsht@163.com (Y.S.); xyrui1201@163.com (Y.X.); fanlu.lab@gmail.com (L.F.); 2 International Joint Research Center of National Animal Immunology, College of Veterinary Medicine, Henan Agricultural University, Zhengzhou 450046, China; zyh7125@163.com (Y.Z.); mengzekun11@163.com (Z.M.)

**Keywords:** avian influenza virus, N9 subtype, neuraminidase, monoclonal antibodies, lateral flow immunoassay

## Abstract

The emergence of highly pathogenic avian influenza viruses (AIVs) poses significant threats to global public health and the poultry economy. Rapid detection methods targeting the hemagglutinin (HA) protein have become increasingly unreliable due to ongoing antigenic drift and genetic mutations. In this study, we screened two monoclonal antibodies (mAbs) against the highly conserved neuraminidase (NA) protein, which were characterized by high affinity and specificity for the N9 subtype. Using the paired mAbs 12A2 and 12F12, an NA-targeting lateral flow immunoassay (NA-LFIA) was developed for the rapid detection of N9 subtype AIVs. The NA-LFIA demonstrated exceptional specificity, exhibiting no cross-reactivity with the N1, N2, or N6 subtypes. The limits of detection (LODs) were established at 10^3.3^ TCID_50_/0.1 mL for the virus and 98 ng/mL for the recombinant NA protein. The test strips exhibited excellent reproductivity for detecting the NA antigen, with intra-assay and inter-assay variations of 5.32% and 6.05%, respectively. Stability testing confirmed a six-month shelf life without any loss of sensitivity. These finds indicate that the NA-LFIA is a robust, rapid point-of-care testing (POCT) tool for the early detection of N9 subtype AIV infection.

## 1. Introduction

The emergence of highly pathogenic avian influenza viruses (AIVs) continues to pose a significant global threat to public health and the poultry economy due to their widespread circulation and high mortality rates in birds [1,2,3]. AIVs possess an exceptionally broad host range, infecting humans as well as numerous species of mammals and birds. To date, 19 HA (H1–H19) and 11 NA (N1–N11) subtypes have been identified in birds or bats, with highly pathogenic influenza viruses predominantly distributed within the H5 and H7 subtypes [4]. In 2013, the novel H7N9 AIV emerged as a triple reassortant of H7, N9, and H9N2 viruses, causing five epidemic waves of human infection in China between 2013 and 2017 [5,6]. The application of the bivalent H5/H7 inactivated vaccine with updated HA genes in poultry has successfully prevented human infections with H7N9 virus [7]. However, the H7N9 AIV remains prevalent in poultry and undergoes rapid evolution on vaccinated farms [8,9].

AIVs, belonging to the *Orthomyxoviridae* family, are enveloped RNA viruses containing eight single-stranded, negative-sense RNA segments. These segments encode several viral proteins, including polymerase subunits (PB2, PB1, PA), hemagglutinin (HA), nucleoprotein (NP), neuraminidase (NA), matrix proteins (M1, M2), and non-structural proteins (NS1, NS2) [10]. The HA and NA proteins are the major envelope glycoproteins of the influenza virus; they play pivotal roles in viral replication and serve as the primary targets of protective immune response [11,12,13]. NA is the second-most abundant glycoprotein, accounting for approximately 10–20% of the total glycoproteins on the virion surface [14,15]. It forms a homotetramer on the viral surface, yielding approximately 40–50 NA spikes on an average-sized virion of 120 nm [14]. Each NA monomer consists of four domains: a membrane distal head, a membrane proximal stalk, a transmembrane domain, and a cytoplasmic tail. Compared to the HA protein, NA typically undergoes less antigenic drift [14,15]. The catalytic site (comprising direct sialic acid contact residues and framework residues) and the second sialic acid-binding site (2SBS) in the NA head domain are highly conserved among AIVs, making them prime targets for antiviral drugs and monoclonal antibody (mAb) development [15,16,17].

Effective detection methods are critical for the prevention, diagnosis, and monitoring of AIV infections. Various laboratory-based diagnostic techniques have been developed, including molecular assays such as reverse transcription–polymerase chain reaction (RT-PCR) and real-time RT-PCR, as well as serological tests like hemagglutination inhibition (HI) and enzyme-linked immunosorbent assay (ELISA). These methods are regularly updated in guidelines provided by the World Health Organization (WHO), the World Organization for Animal Health (WOAH), and the Centers for Disease Control and Prevention (CDC) [18,19,20]. While traditional laboratory-based techniques are highly accurate, they are often time-consuming and may not be feasible in emergency situations or resource-limited areas [19]. Rapid influenza diagnostic tests (RIDTs) are antigen-based tests designed for the rapid point-of-care testing (POCT) of influenza virus infections [21]. Several FDA-approved RIDTs that detect and differentiate between AIV and influenza B virus are currently available on the market; however, none can distinguish between different AIV subtypes [20,21,22]. Recently, immunochromatographic strips based on colloid gold, quantum dot, or time-resolved fluorescence microsphere have been developed for the rapid and sensitive detection of H7 AIVs using HA-targeting mAbs [23,24,25].

In this study, two N9-specific mAbs with high affinity were screened from mice immunized with an inactivated H7N9 virus, utilizing the recombinant N9 NA protein. Using these paired mAbs, an NA-targeting lateral flow immunoassay (NA-LFIA) was developed and characterized for the rapid detection of N9 subtype AIVs.

## 2. Materials and Methods

### 2.1. Viruses

The inactivated H7N9 AIVs, including A/chicken/Guangdong/G1/2013, A/chicken/Guangdong/SW154/2015 and A/chicken/Huizhou/HZ-3/2016, as well as other subtype AIVs of H1N1 A/swine/Guangxi/NN1994/2013, H3N2 A/swine/Guangxi/NNXD/2016, H5N6 A/duck/Yunnan/YN-9/2016, and H9N2 A/chicken/Guangdong/V/2008, were provided by South China Agricultural University. The inactivated H7N9 AIVs including A/chicken/Zhejiang/YH/2/2013 and A/chicken/Guangxi, as well as other subtype AIVs of H11N9 A/duck/Memphis/546/1974, H9N2 A/chicken/Zhejiang/ZS1/2022, H6N6 A/chicken/Zhejiang/ZJ49/2021 and H10N7 A/chicken/Germany/N/1949, were provided by Zhejiang University. The inactivated H7N9 AIVs, including A/chicken/Liaoning/SD005/2020 and A/chicken/Shandong/SD020/2021, as well as the vaccine donor strains of A/pigeon/Shanghai/S1069/2013 (H7-Re1), A/chicken/Guangxi/SD098/2017 (H7-Re2), A/chicken/Inner Mongolia/SD010/2019 (H7-Re3), and A/chicken/Yunnan/SD024/2021 (H7-Re4), were provided by the State Key Laboratory of Veterinary Biotechnology, Harbin Veterinary Research Institute, Chinese Academy of Agricultural Sciences. The avian viruses, including Newcastle disease virus (NDV), infectious bursal disease virus (IBDV), infectious bronchitis virus (IBV), and Marek’s disease virus (MDV), were provided by the Institute for Animal Health.

### 2.2. Generation of MAbs

MAbs against H7N9 AIV were developed following a standard procedure as previously described [24]. Briefly, six-week-old female BALB/c mice were immunized with the inactivated H7N9 AIV (A/chicken/Guangdong/G1/2013) emulsified with Freund’s adjuvant at 20 μg/mouse three times at a 2-week interval. Two weeks after the third immunization, serum samples were collected from the immunized mice and the antibody titers were determined by ELISA. The mouse with the highest antibody titer was then selected for booster immunization at a dose of 50 μg three days before cell fusion. Splenocytes from the immunized mouse were fused with Sp2/0 myeloma cells at a ratio of 1:2. The hybridoma cells were screened by ELISA and the immunoperoxidase monolayer assay (IPMA) followed by limiting dilution cloning.

### 2.3. Characterization of MAbs Targeting NA Protein

#### 2.3.1. ELISA Screening

ELISA was performed to screen mAbs against the N9 NA protein of AIV and determine antibody titers. The H7N9 recombinant NA protein (Sino Biological Inc., Beijing, China) diluted in carbonate buffer (CBS, pH 9.6) at a concentration of 1 μg/mL was added into 96-well plates at 100 μL/well and incubated at 37 °C for 2 h. After blocking with 5% skim milk at 37 °C for 1 h, hybridoma supernatants or serially diluted ascites were added and incubated at 37 °C for 30 min. The HRP-conjugated goat anti-mouse IgG diluted at 1:5000 was then added to detect the bound mAbs. Color was developed with 3,3′,5,5′-Tetramethylbenzidine (TMB) substrate solution at room temperature (RT) for 10 min followed by adding a stop solution, and absorbance was read at 450 nm with a microplate reader (Flash Spectrum, Shanghai, China) for statistical analysis.

#### 2.3.2. Western Blot Analysis

Western blot was performed to further verify the reactivity of the anti-NA protein mAbs. Briefly, 20 μL of the purified viral protein, which is mixed with loading buffer and boiled for 10 min, was applied to a 10% SDS-PAGE gel. After electrophoresis, the separated proteins were transferred to a nitrocellulose membrane followed by blocking with 5% skim milk. The membrane was then incubated subsequently with mAb supernatants and horseradish peroxidase (HRP)-conjugated goat anti-mouse IgG as the primary and secondary antibodies, respectively. The results were identified with an enhanced chemiluminescent (ECL) substrate (NCM Biotech, Suzhou, China).

#### 2.3.3. MAb Characterization

The subtypes of the mAbs specific for NA protein were identified using a mouse mAb subtype identification kit (Proteintech, Wuhan, China). Antibody binding analysis was carried out using a surface plasmon resonance (SPR) assay. The specificity of mAbs against NA protein was determined by indirect immunofluorescence assay (IFA) using the HEK293T cells transfected with the NA recombinant plasmid.

### 2.4. Colloidal Gold Conjugation of MAbs

#### 2.4.1. Colloidal Gold Preparation

Colloidal gold nanoparticles (AuNPs) were prepared by the trisodium citrate method [26]. Briefly, the Erlenmeyer flask containing 99 mL of double-distilled water was filled with 1 mL of 1% chloroauric acid while being stirred and heated. Subsequently, 1.6 mL of 1% trisodium citrate solution was added quickly while stirring. The mixture was further heated for 5 min, and then gradually boiled until the color changed from light yellow to deep crimson and stabilized. After cooling to RT, the AuNPs were stored at 4 °C.

#### 2.4.2. Conjugation of MAbs

The saturation concentration of mAbs for AuNPs was measured before colloidal gold conjugation. In a dilution plate, 1 μL of mAbs was added into 10 μL dH_2_O followed by a serial two-fold dilution. Then, 125 μL of pH-adjusted AuNPs was added to the diluted mAb solution and incubated at RT for 5 min. After adding 125 μL of 10% sodium chloride, the mAb saturation on AuNPs was determined by observing the resulting color change. To prepare the AuNP-mAb conjugates, the mAbs at the optimized concentration were incubated with colloidal gold solution at RT for 30 min. After adding 100 μL of 10% bovine serum albumin (BSA), the conjugation mixture was incubated for an additional 10 min. To remove unbound antibodies, the mixture was centrifuged at 12,000 r/min for 20 min. Finally, the resulting pellet was suspended in a borate buffer containing 1% BSA, 3% trehalose and 0.03% sodium azide.

### 2.5. Selection of Paired MAbs by Sandwich Dot-Blot

Two mAbs with higher binding affinity to the N9 NA protein of AIV were selected for LFIA by sandwich dot-blot. Briefly, nine different mAbs were spotted onto each nitrocellulose (NC) membrane in a 3 × 3 array to serve as capture antibodies (Table 1) and dried at 37 °C for 30 min. After being blocked with 1% BSA, the membranes were incubated with 200 μL of the inactivated H7N9 viruses at RT for 30 min followed by washing with Phosphate-buffered saline (PBS) containing 0.2% Tween 20. Then 200 μL of the nine mAbs conjugated with AuNPs were applied to the antigen-captured membrane respectively and incubated at RT for 30 min to detect the captured antigens. After the unbound AuNps were removed by washing, the paired capture and conjugate mAbs showing the strongest colored dot on the membrane were selected for the test line and conjugation.

### 2.6. Preparation of the Test Strip

**Figure 1:**
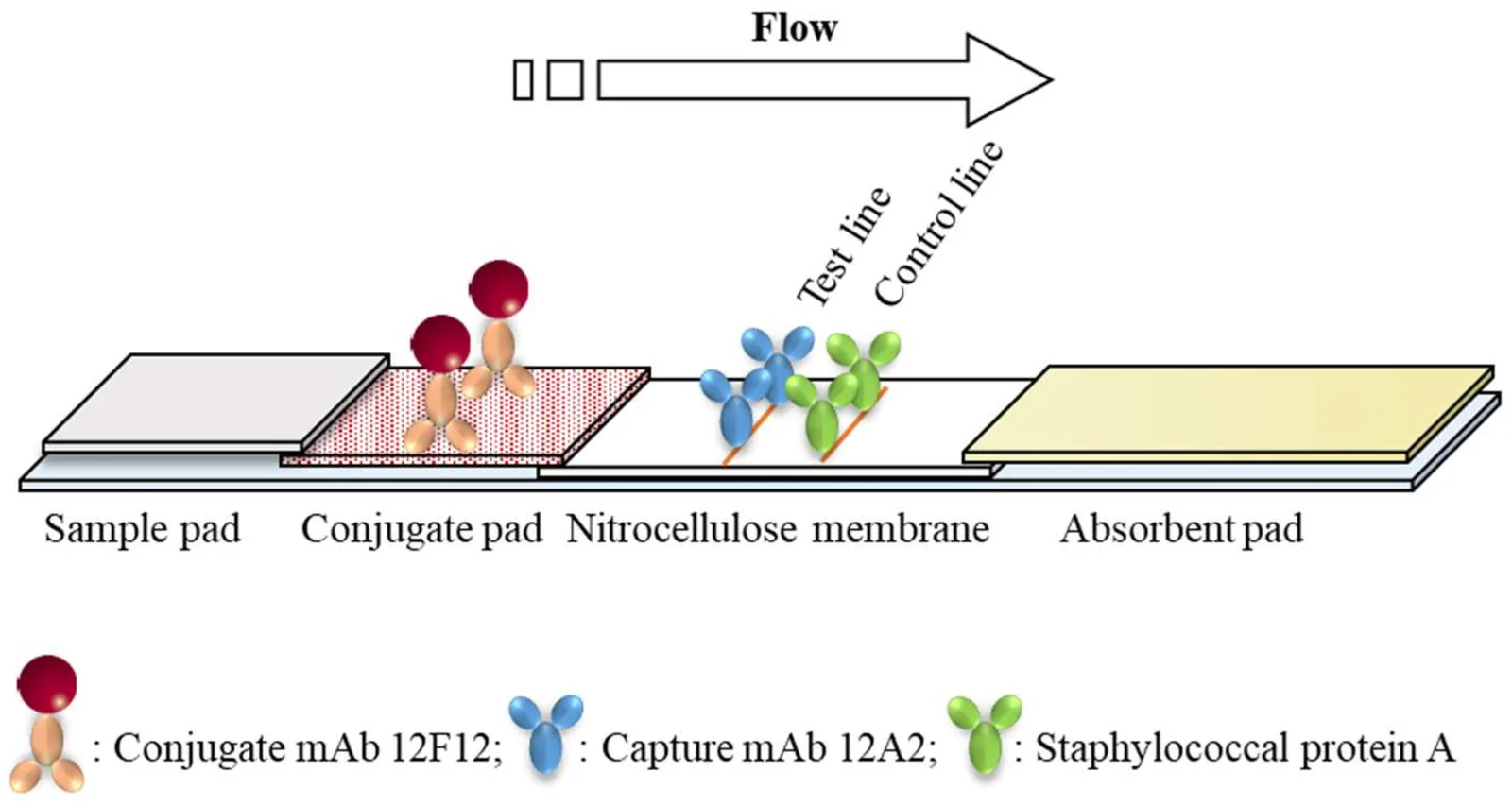
Schematic illustration of the NA-LFIA strip assembly. The test strip comprises a sample pad, a conjugate pad loaded with AuNP(red)-labeled mAb 12F12 (targeting N9 NA, orange), a nitrocellulose membrane immobilized with the paired mAb 12A2 (blue) as the test line and Staphylococcal protein A (SPA, green) as the control line, and an absorbent pad on a backing card.

The NA-specific capture mAb and staphylococcal protein A (SPA) solution were dispensed as test and control lines on the NC membrane, respectively. The blotted membrane was then dried at 45 °C for 4 h. To prepare the conjugate pad, the paired NA mAb was conjugated with AuNPs and distributed on the fiberglass pads, which were dried at 42 °C for 50 min. The absorbent pad, nitrocellulose membrane, conjugate pad, and sample pad were assembled on the backing card sequentially to produce the immunochromatographic test strip (Figure 1).

### 2.7. Determination of NA-LFIA Broad-Spectrum Reactivity

N9 subtype AIV isolates from different places as well as vaccine donor strains of H7-Re1, -Re2, -Re3, and -Re4 were tested by the N9 test strips respectively to assess broad-spectrum reactivity of the NA-LFIA. In the LFIA test, 100 μL of sample containing 10^5^ TCID_50_ viruses or a 10-fold diluted antigenic strain was added onto the sample pad and incubated at RT for 10 min.

### 2.8. Specificity Evaluation of NA-LFIA

N1, N2, N6, N7, and N9 subtype AIVs and other avian viruses such as Newcastle disease virus (NDV), infectious bursal disease virus (IBDV), infectious bronchitis virus (IBV), and Marek’s disease virus (MDV) were simultaneously detected using the test strips to evaluate specificity of the NA-LFIA.

### 2.9. Determination of NA-LFIA Sensitivity

The H7N9 AIV of A/chicken/Guangdong/G1/2013 and H7N9 recombinant NA protein were used to determine sensitivity of the LFIA. The inactivated H7N9 virus (10^5.7^ TCID_50_/0.1 mL) and NA protein (50 µg/mL) were serially two-fold diluted from 2^−1^ to 2^−11^ and 2^−1^ to 2^−10^ with 0.01 M PBS, respectively. Using 0.01 M PBS as a negative control, 100 μL of the diluted samples was detected by the NA-LFIA test strips, respectively.

### 2.10. Reproducibility Evaluation of NA-LFIA

The reproducibility of the NA-LFIA was evaluated using the recombinant NA protein. Intra-assay precision (within-batch) was determined by testing five parallel replicates of NA protein samples at concentrations of 25, 5, and 1 µg/mL using a single batch of test strips. Inter-assay precision (between-batch) was assessed across three independent batches of test strips. The test strips were scanned using an LFIA strip reader (CSY-JAA, Shenzhen, China) to measure the optical density (OD) of the test line, which was subsequently used to calculate the coefficient of variation (CV).

### 2.11. Stability Evaluation of NA-LFIA Test Strip

After being stored at 4 °C for 0, 3 and 6 months, the sensitivity and specificity of the test strip were determined as described above.

### 2.12. Clinical Applications of NA-LFIA Test Strips

The respiratory tract tissues (including trachea and lungs) were collected from six experimentally infected chickens and two uninfected chickens as described previously [24]. Additionally, 50 oropharyngeal swabs were collected from poultry farms in Luoyang, China, which were placed in PBS containing 1% formaldehyde. Subsequently, these samples were tested in parallel using NA-LFIA test strips and RT-PCR.

## 3. Results

### 3.1. Selection of Paired mAbs for the NA-LFIA

**Figure 2:**
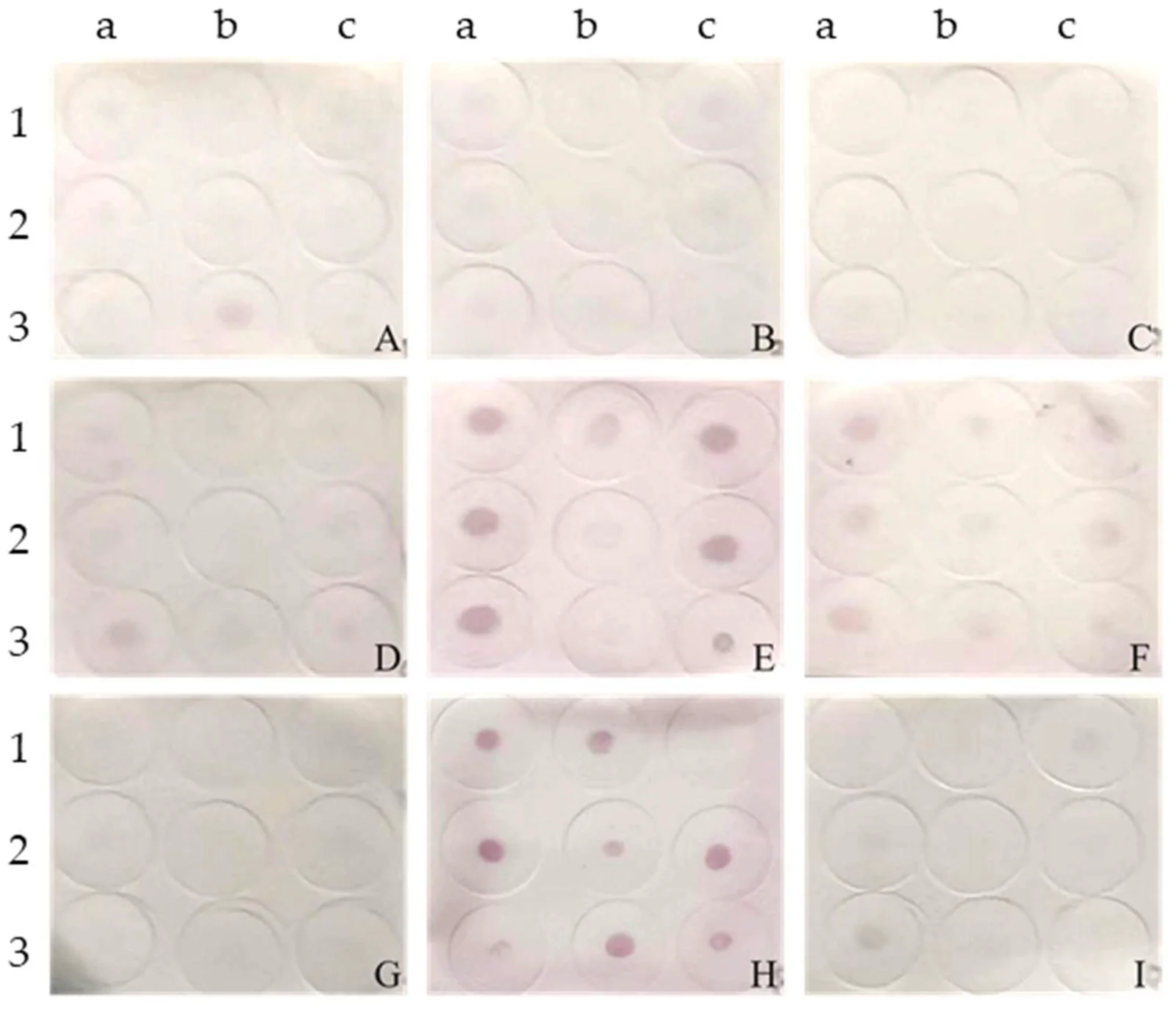
Screening of antibody pairs by sandwich dot-blot. Replicate nitrocellulose membrane strips, each printed with a 3 × 3 array of the capture mAbs (Table 1), were incubated with inactivated H7N9 virus. Antigen capture was detected using individual AuNP-labeled mAbs: 1D4 (**A**), 6H10 (**B**), 6G7 (**C**), 12A2 (**D**), 2E6 (**E**), 2E2 (**F**), 6C7 (**G**), 12F12 (**H**), and 4D10 (**I**).

Nine mAbs specific for the N9 NA protein were successfully generated from mice immunized with the H7N9 AIV (Figure S1). To identify optimal capture and conjugate mAbs for the NA-LFIA, these 9 mAbs were blotted onto a nitrocellulose membrane, incubated with the inactivated H7N9 virus, and subsequently detected using the AuNP-labeled mAbs. Clear, distinct colored dots were observed on the membrane when detected by the conjugate mAb 12F12 (Figure 2). Among these dots, mAb 12A2 exhibited the highest color intensity (Figure 2H), indicating mAbs 12A2 and 12F12 function as the best paired antibodies for capture and conjugation, respectively.

### 3.2. Characteristics of the Paired MAbs

**Figure 3:**
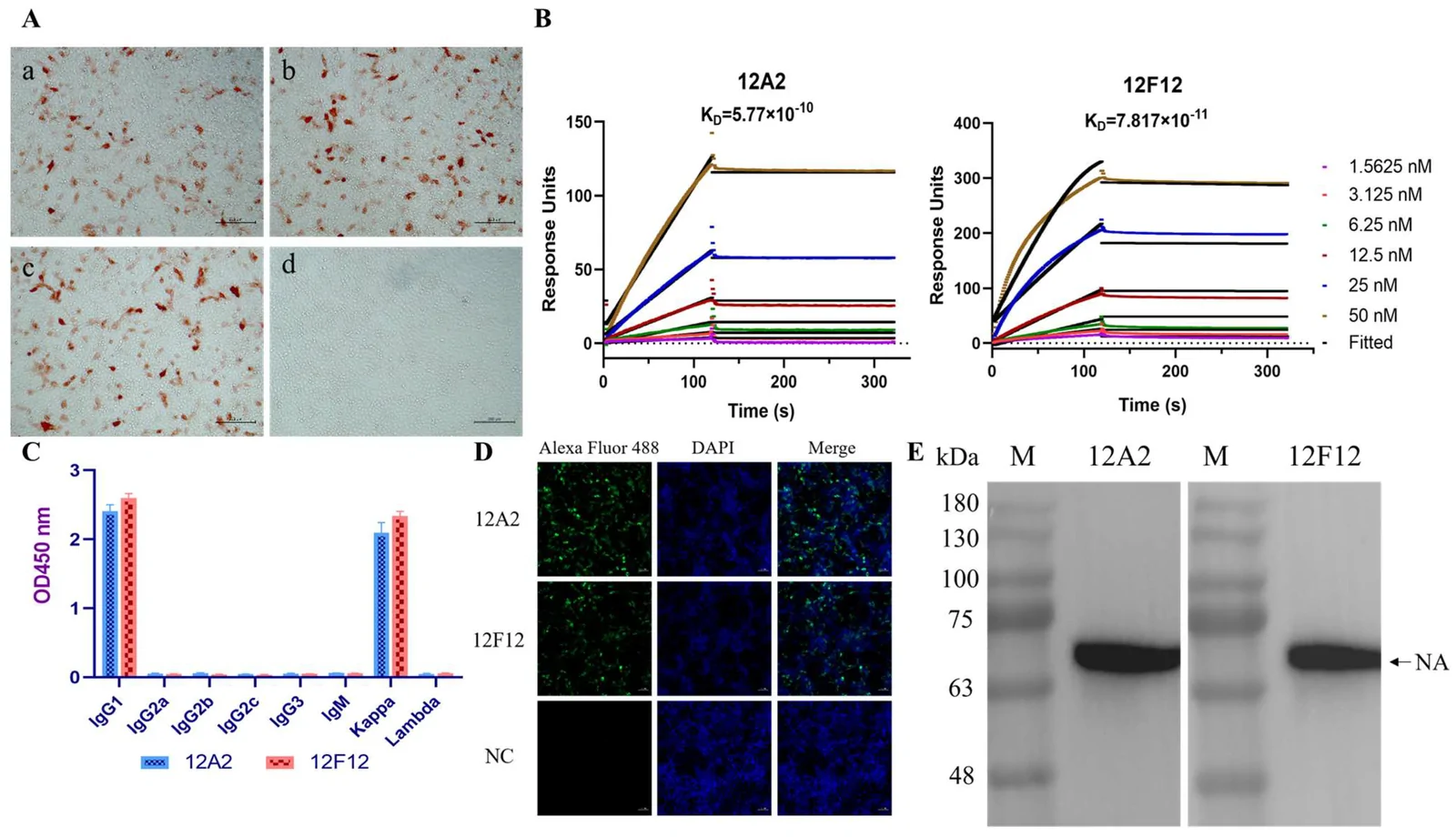
Characterization of N9 NA-specific mAbs. (**A**) IPMA validation on H7N9 AIV-infected cells: (**a**) 12A2, (**b**) 12F12, (**c**) positive control serum, (**d**) negative control serum. Scale bars, 200 μm. (**B**) Representative SPR binding kinetics of NA protein to mAbs 12A2 and 12F12. Affinity constants (*K_D_*) were determined using Biacore X100 Evaluation Software (version 2.0.2); data represent mean ± SD (*n* = 3). (**C**) Mab isotyping by ELISA. (**D**) IFA of mAbs 12A2 and 12F12 targeting transiently expressed NA protein in HEK293T cells: NA protein (green), nuclei (blue); NC, negative control. Scale bars, 50 μm. (**E**) Western blot showing mAb reactivity against purified viral protein. Lane M, Protein Marker.

Two mAbs of 12A2 and 12F12 targeting the N9 NA protein were selected for LFIA development to detect N9 AIVs. The antibody titers for both mAbs were determined to be 10^−6^ via IPMA (Figure 3A). Furthermore, SPR assays revealed K_D_ values of 5.77 × 10^−10^ M for 12A2 and 7.82 × 10^−11^ M for 12F12, demonstrating that both mAbs possess an exceptionally high affinity for the NA protein (Figure 3B). The mAbs were isotyped as IgG1 with a κ light chain via ELISA (Figure 3C). They exhibited strong reactivity and high specificity toward the NA protein expressed in the transfected cells (Figure 3D), and specifically recognized the viral NA protein at approximately 70 kDa in Western blot analyses (Figure 3E).

### 3.3. Production of the NA-LFIA Test Strip

Based on the paired mAbs specific for N9 NA protein, the mAb 12F12 conjugated with colloidal gold was dispensed on the fiberglass pads. The paired mAb 12A2 and SPA were dispensed on the NC membrane as the test and control lines respectively. The absorption pad, NC membrane, antibody-labeled conjugate pad, and fiberglass sample pad were then assembled to produce the test strip for detecting H7N9 AIVs.

### 3.4. Broad Reactivity of the NA-LFIA Test Strip

**Figure 4:**
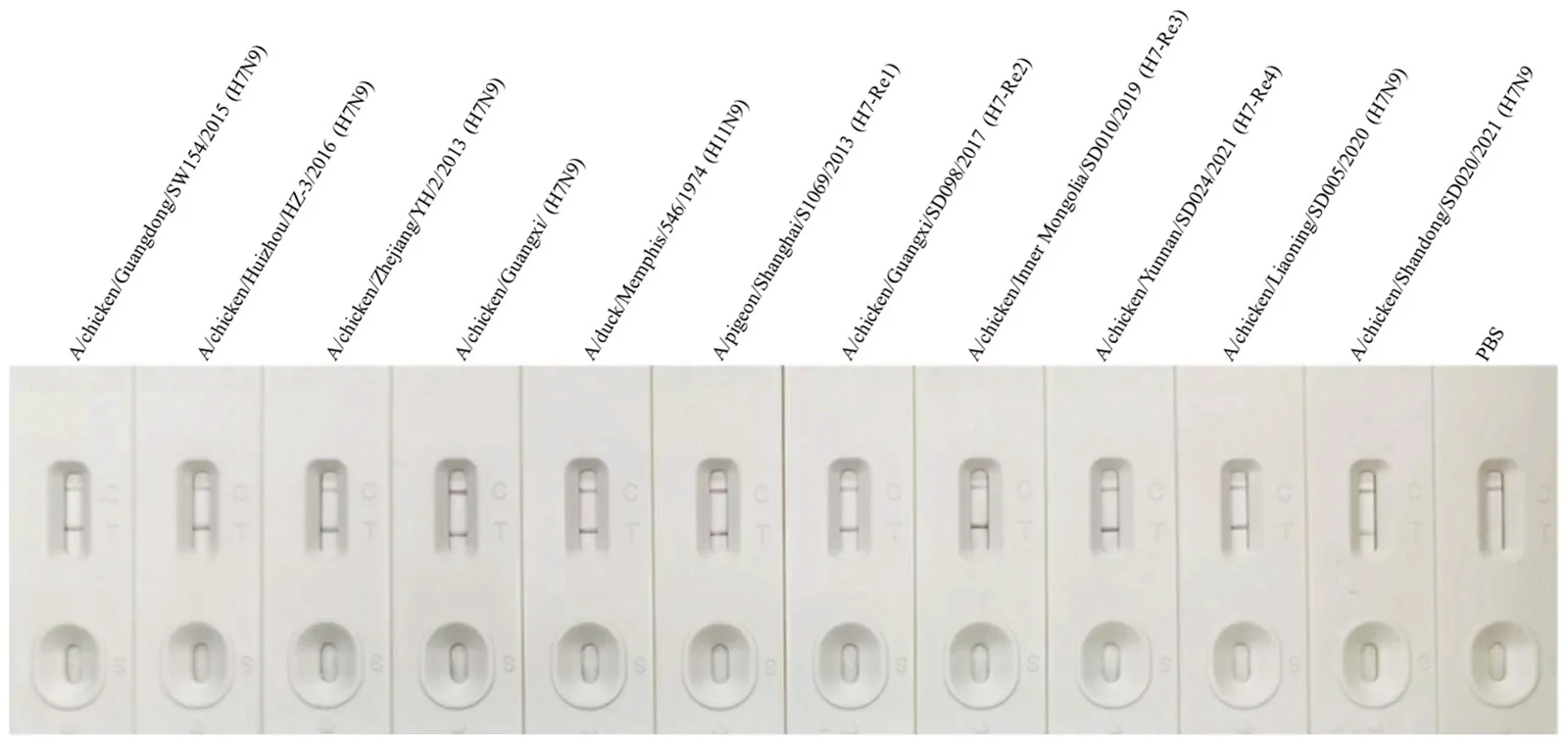
Broad-spectrum reactivity of the NA-LFIA test strip against N9 subtype AIVs. Six inactivated H7N9 strains isolated from distinct locations and time periods, alongside an H11N9 isolate and vaccine donor strains (H7-Re1, H7-Re2, H7-Re3, and H7-Re4), were detected using the test strips. PBS was used as the negative control.

All tested N9 subtype AIVs (H7N9 and H11N9) and main antigenic components in inactivated vaccines, including H7-Re1, Re-2, Re-3, and Re-4 antigen strains, showed two red lines on the detection membrane (Figure 4 and Table S1), indicating that the NA-LFIA test strip had a broad reactivity for detecting N9 subtype AIVs.

### 3.5. Specificity of the LFIA Test Strip

**Figure 5:**
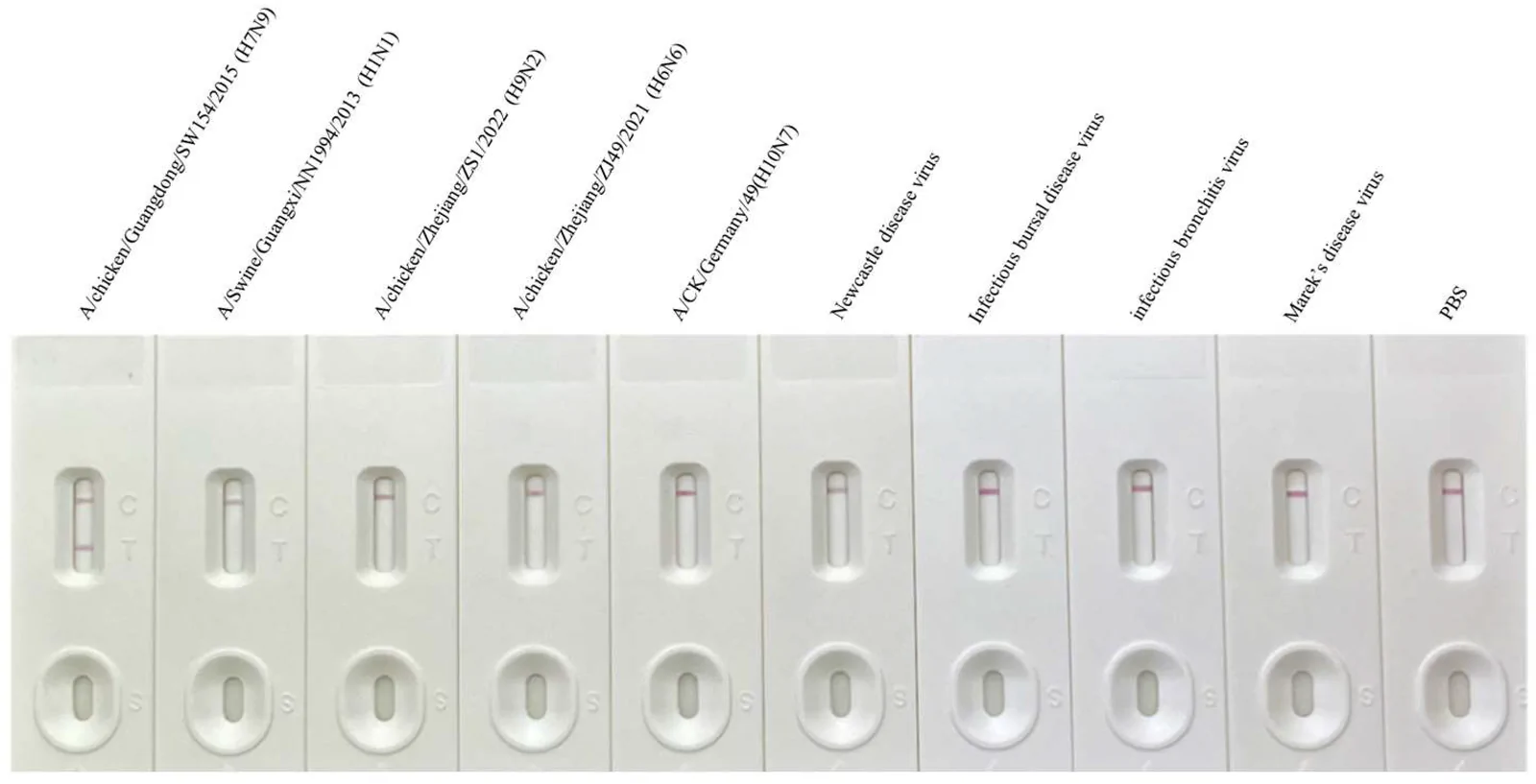
Specificity of the NA-LFIA test strip. Non-target AIV subtypes (H9N2, H6N6, and H10N7), along with other common avian viruses, including Newcastle disease virus (NDV), infectious bursal disease virus (IBDV), infectious bronchitis virus (IBV), and Marek’s disease virus (MDV), were detected by the test strips. H7N9 AIV and PBS were used as the positive and negative controls, respectively.

The specificity test results showed that only N9 subtype AIVs had two red bands at the T and C lines; N1, N2, N6, N7, and other poultry viruses showed only one red band at the C line (Figure 5 and Table S1), indicating that the strip had high specificity in detecting N9 subtype AIVs.

### 3.6. Sensitivity of the NA-LFIA Test Strip

**Figure 6:**
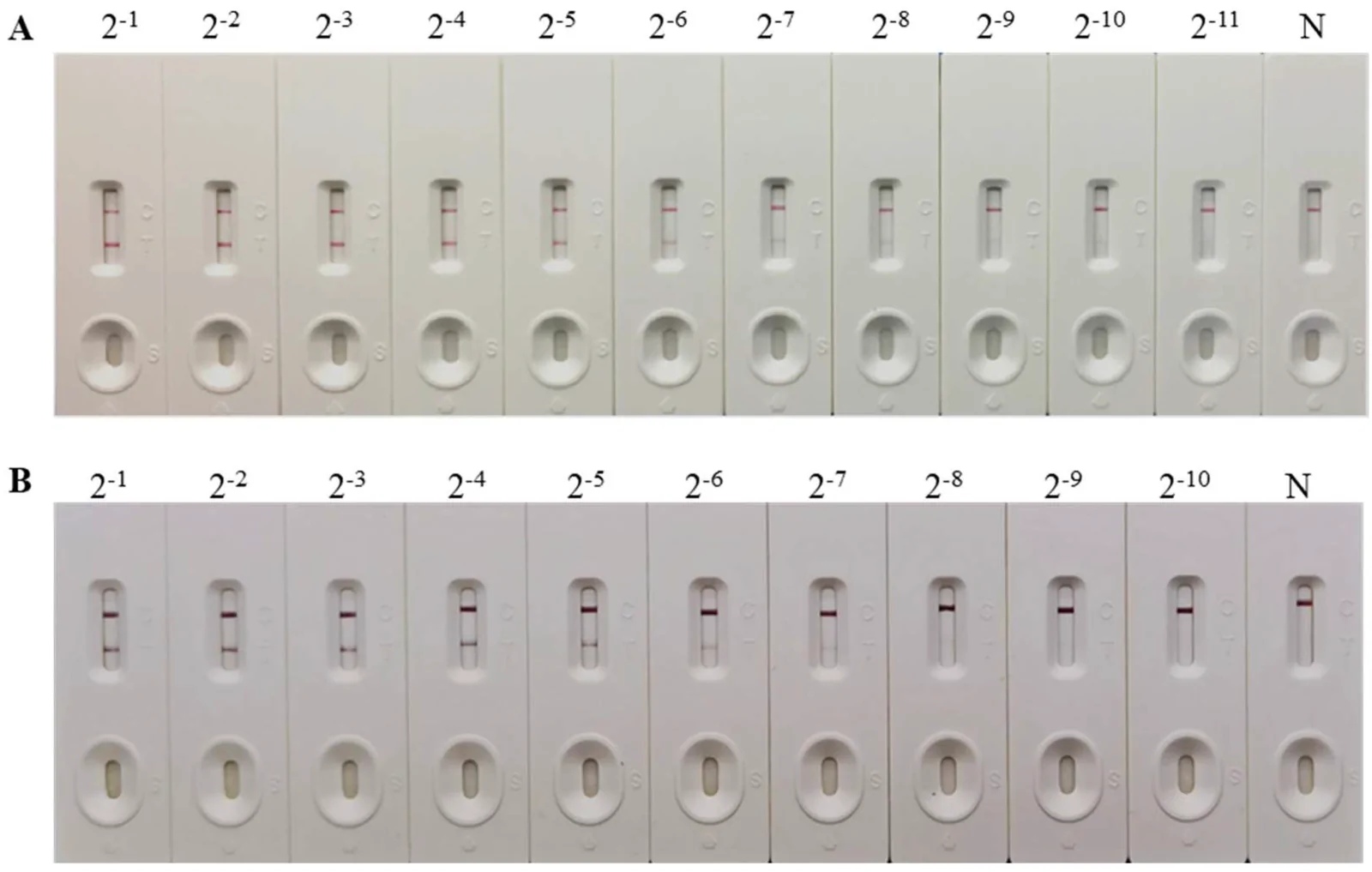
Sensitivity of the NA-LFIA test strip. The inactivated H7N9 AIV A/chicken/Guangdong/G1/2013 (10^5.7^ TCID_50_/0.1 mL) diluted from 2^−1^ to 2^−11^ (**A**) and the H7N9 recombinant NA protein (50 µg/mL) diluted from 2^−1^ to 2^−10^ (**B**) were detected by the NA-LFIA test strips using PBS as a negative control (N), respectively.

The sensitivity of the NA-LFIA was evaluated using the H7N9 AIV strain A/chicken/Guangdong/G1/2013 and the recombinant NA protein. The color intensity of the test lines progressively diminished as the viral titer or NA protein concentration decreased. Both the H7N9 AIV antigen and the NA protein showed positive detection results at dilutions as low as 2^−9^ (1:512) (Figure 6). Consequently, the LODs of the NA-LFIA were calculated to be 10^3.3^ TCID_50_/0.1 mL for the H7N9 AIV and 98 ng/mL for the NA protein.

### 3.7. Reproducibility of the NA-LFIA Test Strip

Analytical precision assessments demonstrated consistent performance metrics for the NA-LFIA test strip. The average intra-assay CV was 5.32% within the same batch, whereas the inter-assay CV across three independent batches was 6.05% (Table 2). This indicates high reproducibility and robust technical assembly of the NA-LFIA platform.

### 3.8. Stability of the NA-LFIA Test Strip

After six months of storage, the NA-LFIA test strip detected specifically as low as 1:512 (2^−9^) diluted H7N9 AIV (A/chicken/Guangdong/G1/2013) with no cross-reactivity with the other related viruses (Table 3), indicating that the test strip had high stability for detecting H7N9 AIVs.

### 3.9. Detection of Clinical Samples

**Figure 7:**
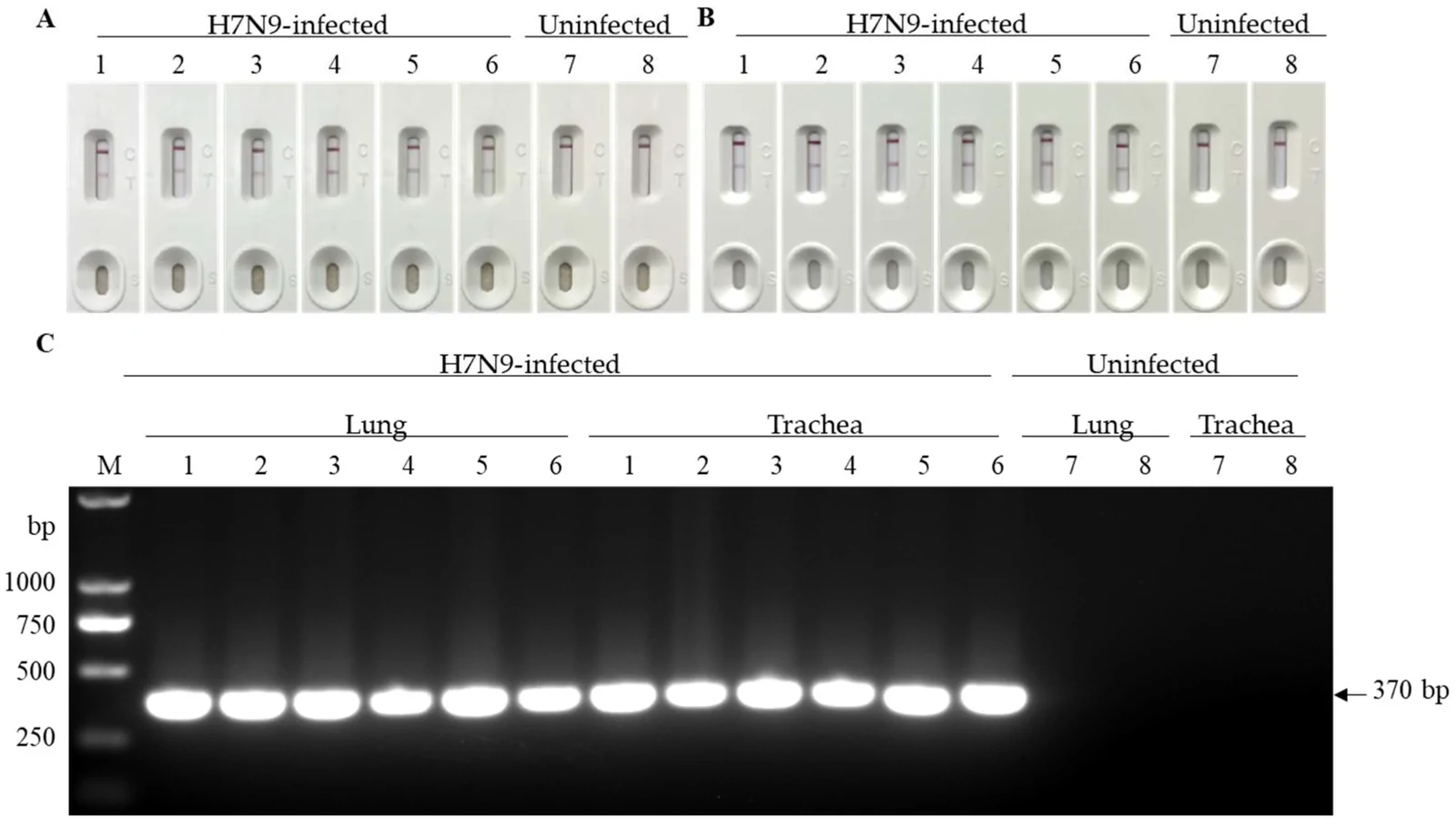
Evaluation of the NA-LFIA on clinical tissue matrices from H7N9-infected chickens. Lung (**A**) and trachea (**B**) samples collected from six experimentally H7N9-infected chickens (1–6) and two uninfected healthy chickens (7 and 8; negative controls) were evaluated using the test strips. (**C**) validation of all lung and trachea samples by RT-PCR as the reference standard.

Trachea and lung samples from eight chickens, comprising six H7N9-infected chickens and two uninfected controls, were evaluated using both RT-PCR and NA-LFIA test strips. The NA-LFIA successfully detected the H7N9 AIV antigen in 100% (12/12) of the infected tissue samples and in none (0/4) of the uninfected tissues, demonstrating complete concordance with the RT-PCR results (Figure 7). Additionally, no H7N9 AIV antigen or RNA was detected by either the NA-LFIA or RT-PCR in clinical swab samples evaluated.

## 4. Discussion

NA is the second-most abundant surface glycoprotein of influenza viruses and acts as a crucial receptor-destroying enzyme across multiple stages of the virus life cycle [15,16,17]. The NA active site remains highly conserved due to the functional constraints associated with sialic acid recognition [14]. Compared to the HA protein, NA typically undergoes slower antigenic drift, which is generally discordant with the evolutionary trajectory of HA [27]. Consequently, anti-NA antibodies potentially offer broader cross-reactivity against heterologous strains than anti-HA antibodies, underscoring their value as a supplementary approach for AIV prevention [15]. Several broadly neutralizing antibodies targeting the NA protein, such as the human-derived mAb 1G01, have been shown to cross-react with various AIV subtypes by directly binding to these conserved catalytic sites [28,29,30]. Recently, a pan-influenza NA-targeting mAb that inhibits neuraminidase via receptor mimicry was reported, displaying unprecedented breadth and potency against both influenza A and B viruses [31]. In this study, we successfully produced and characterized 9 mAbs specific for N9 NA protein by immunizing mice with an inactivated H7N9 AIV. Two of these antibodies (12A2 and 12F12) bound with low nanomolar affinities to the N9 NA protein. Moreover, both the NA-targeting mAbs demonstrated potent neuraminidase inhibition (NI) activity against the H7N9 AIV, yielding a 50% inhibitory concentration (IC_50_) of 9.77 ng/mL in an enzyme-linked lectin assay (ELLA) (Figure S2). However, no significant neutralizing activity was observed during in vitro virus neutralization assays (Figure S3). This suggests that mAbs 12A2 and 12F12 likely bind proximal to the active site, inhibiting NA activity through steric hindrance rather than by binding directly to epitopes within the enzyme’s active site.

LFIA is a simple, sensitive, and practical diagnostic technique that can be deployed without advanced laboratory infrastructure or biological protection equipment, offering a rapid, early, and large-scale approach for detecting AIV infections [32,33]. Several colloidal gold-based LFIA test strips have previously been developed using H7 HA-specific mAbs for the rapid detection of H7N9 infection, with LODs recorded at 2.5 log_10_EID_50_/0.1 mL [24,34] or 10^3^ TCID_50_/mL [35]. However, H7N9 AIVs continue to undergo accelerated evolution under significant vaccination pressure. Vaccine strains have required regular updates since the introduction of the H7N9-inactivated vaccine (H7-Re1) in 2017, culminating in the replacement of the H7-Re3 strain with H7-Re4 in 2022 [36,37]. Because of the high mutation rate associated with the HA protein, RIDTs relying on HA-targeting LFIAs may fail to detect dominant field strains. In our laboratory, we found that HA-targeting LFIA test strips could detect the H7-Re2 strain but failed to identify H7-Re3 [24]. To overcome this limitation, we developed an NA-targeting LFIA based on high-affinity paired mAbs designed specifically to detect the N9 NA protein. The NA-LFIA demonstrates robust broad-spectrum reactivity with various H7N9 isolates, including the vaccine donor strains H7-Re1, -Re2, -Re3, and -Re4. Notably, the test strip successfully recognized a heterologous H11N9 virus (A/duck/Memphis/546/1974), affirming its broad-spectrum capability as an N9-specific diagnostic tool. The NA-LFIA possesses high sensitivity, with an LOD of 10^3.3^ TCID_50_/0.1 mL, which is comparable to existing HA-targeting strips. Furthermore, the NA-LFIA test strip is highly specific to the N9 subtype and exhibits no cross-reactivity with the N1, N2, or N6 AIV subtypes. Therefore, this NA-targeting LFIA holds great potential as a quick, easy, and reliable POCT technology for the rapid field detection of N9 subtype AIV infections.

Due to the remarkable success of compulsory poultry vaccination programs across China since 2017, the natural field prevalence of H7N9 AIV has declined drastically, which severely constrains the availability of positive clinical field swab samples. While our animal infection model provided a robust alternative for tissue validation, expanding validation utilizing clinical swab samples will serve as the primary focus of our subsequent field studies.

## Figures and Tables

**Table 1:** Dot-blot of the nine mAbs on the nitrocellulose membrane.

mAbs	a	b	c
1	1D4	6H10	6G7
2	12A2	2E6	2E2
3	6C7	12F12	4D10

**Table 2:** Reproducibility evaluation of the NA-LFIA test strip.

NA Protein (µg/mL)	Intra-Assay (*n* = 5)		Inter-Assay (*n* = 3)	
	OD Value	CV	OD Value	CV
25	0.914 ± 0.030	3.34%	0.913 ± 0.031	3.51%
5	0.386 ± 0.018	4.71%	0.393 ± 0.025	6.30%
1	0.142 ± 0.008	7.92%	0.140 ± 0.010	8.33%

**Table 3:** Stability evaluation of the NA-LFIA.

Storage Time (Month)	Sensitivity	Specificity
0	2^−9^ (1:512)	N9-specific
3	2^−9^ (1:512)	N9-specific
6	2^−9^ (1:512)	N9-specific

## Data Availability

The data supporting the conclusions of this article are included within the article. The raw data are available upon request.

## Supplementary Materials

The following supporting information can be downloaded at: https://www.mdpi.com/article/10.3390/v18080879/s1, Figure S1: Screening of mAbs specific for H7N9 AIV by IPMA. A: Negative control; B: 12A2; C: 12F12; D: 2E2; E: 6G7; F: 6C7; G: 2E6; H: 4D10; I: 6H10; J: 1D4, Scale bars, 200 μm; Figure S2: Inhibition of NA enzymatic activity by anti-NA mAbs as measured ELLA; Figure S3: Determination of neutralizing activity of anti-NA mAbs. A: Virus control; B: Positive control; C: 12A2; D: 12F12; E: 2E2; F: 6G7; G: 6C7; H: 2E6; I: 4D10; J: 6H10; K: 1D4, Scale bars, 200 μm; Table S1: Reactivity and specificity of the NA-LFIA test strip against various avian influenza virus (AIV) strains.

## Abbreviations

The following abbreviations are used in this manuscript:

2SBS	The second sialic acid-binding site
AIV	Avian influenza virus
AuNP	Gold nanoparticle
BSA	Bovine serum albumin
CBS	carbonate buffer
CDC	Centers for Disease Control and Prevention
CV	Coefficient of variation
ECL	Chemiluminescent
ELISA	Enzyme-linked immunosorbent assays
ELLA	enzyme-linked lectin assay
FDA	Food and Drug Administration
HA	Hemagglutinin
HI	Hemagglutination inhibition
HRP	Horseradish peroxidase
IBDV	Infectious bursal disease virus
IBV	Infectious bronchitis virus
IFA	Immunofluorescence assay
IC_50_	50% inhibitory concentration
LFIA	Lateral flow immunoassay
LOD	limit of detection
mAb	Monoclonal antibody
MDV	Marek’s disease virus
NA	Neuraminidase
NC	Nitrocellulose
NDV	Newcastle disease virus
NI	Neuraminidase inhibition
OD	Optical density
PBS	Phosphate-buffered saline
POCT	Point-of-care testing
RIDT	Rapid influenza diagnostic test
ROD	Relative optical density
RT	Room temperature
RT-PCR	Reverse transcription–polymerase chain reaction
SPA	Staphylococal protein A
SPR	Surface plasmon resonance
TCID_50_	50% tissue culture infectious dose
TMB	3,3′,5,5′-Tetramethylbenzidine
WHO	World Health Organization
WOAH	World Organization for Animal Health
