# Supplementary material for: Rapid Detection of N9 Subtype Avian Influenza Viruses Using an NA-Targeting Lateral Flow Immunoassay
Source: Viruses. 2026 Aug 11;18(8):879. doi: 10.3390/v18080879 (PMC13517291; doi:10.3390/v18080879)
Supplement: Supplementary file 1 [file viruses-18-00879-s001.zip › viruses-4410627-supplementary.pdf]

viruses

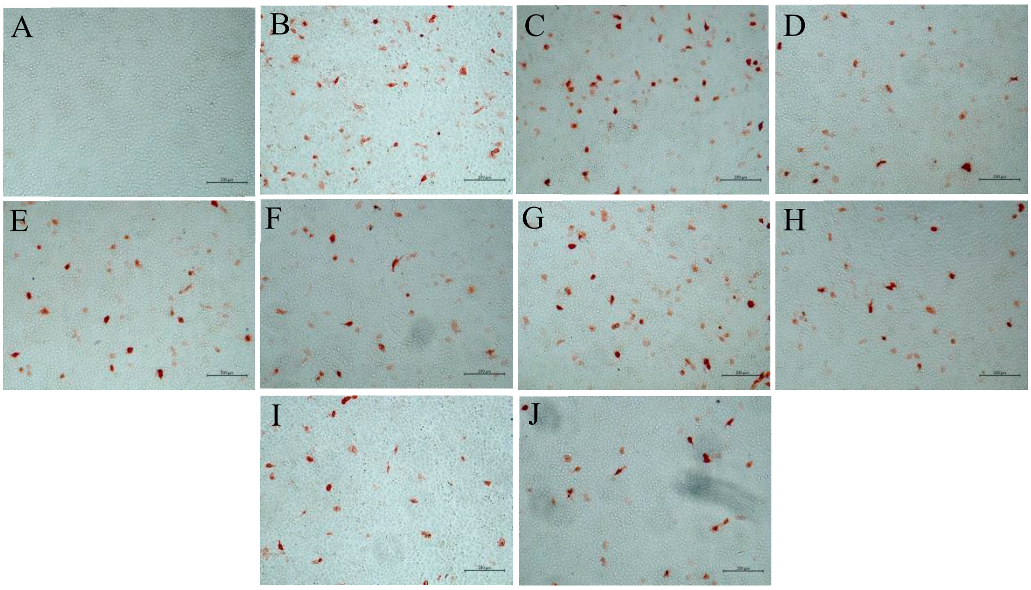

Figure S1. Screening of mAbs specific for H7N9 AIV by IPMA assay. A: Negative control; B: 12A2; C: 12F12; D: 2E2; E: 6G7; F: 6C7; G: 2E6; H: 4D10; I: 6H10; J: 1D4. Scale bars, 200 μm.

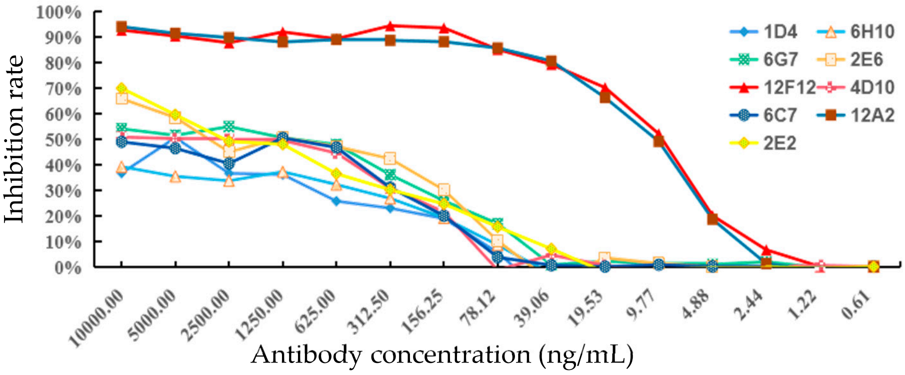

Figure S2. Inhibition of NA enzymatic activity by anti-NA mAbs as measured ELLA.

 https://doi.org/10.3390/v18080879

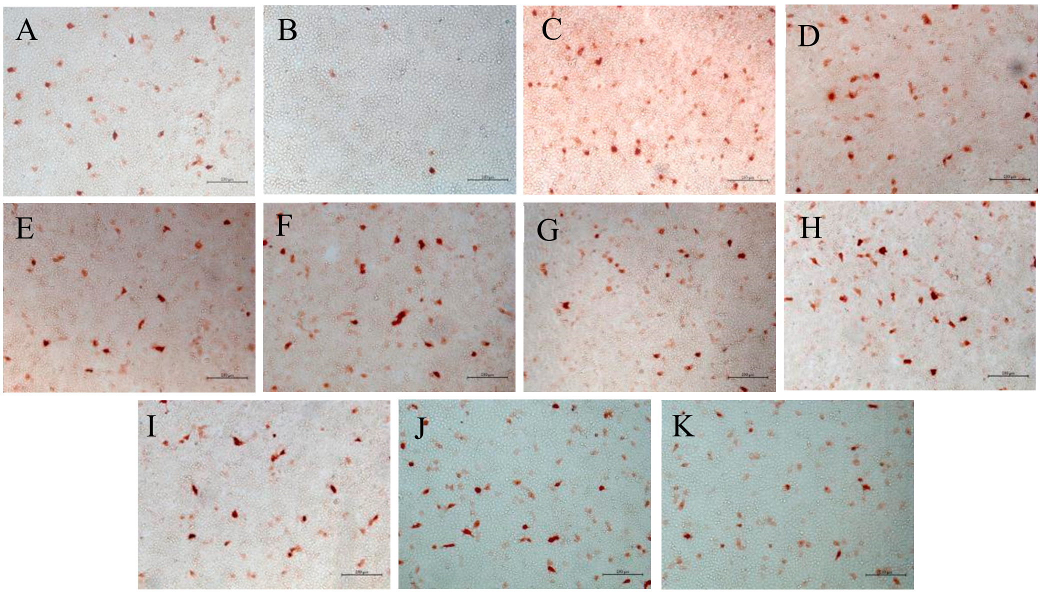

Figure S3. Determination of neutralizing activity of anti-NA mAbs. A: Virus control; B: Positive control; C: 12A2; D: 12F12; E: 2E2; F: 6G7; G: 6C7; H: 2E6; I: 4D10; J: 6H10; K: 1D4. Scale bars, 200 μm.

Table S1. Reactivity and specificity of the NA-LFIA test strip against various avian influenza virus (AIV) strains

No.	Isolates	Subtype	Year	Reactivity	Donor strains
1	A/chicken/Guangdong/G1/2013	H7N9	2013	+	
2	A/chicken/Zhejiang/YH/2/2013	H7N9	2013	+	
3	A/chicken/Guangdong/SW154/2015	H7N9	2015	+	
4	A/chicken/Huizhou/HZ-3/2016	H7N9	2016	+	
5	A/chicken/Liaoning/SD005/2020	H7N9	2020	+	
6	A/chicken/Shandong/SD020/2021	H7N9	2021	+	
7	A/chicken/Guangxi	H7N9	/	+	
8	A/pigeon/Shanghai/S1069/2013	H7N9	2013	+	H7-Re1
9	A/chicken/Guangxi/ SD098/2017	H7N9	2017	+	H7-Re2
10	A/chicken/Inner Mongolia/SD010/2019	H7N9	2019	+	H7-Re3
11	A/chicken/ Yunnan/SD024/2021	H7N9	2021	+	H7-Re4
12	A/duck/Memphis/546/1974	H11N9	1974	+	
13	A/swine/Guangxi/NN1994/2013	H1N1	2013	-	
14	A/swine/ Guangxi/NNXD/2016	H3N2	2016	-	
15	A/chicken/ Guangdong/V/2008	H9N2	2008	-	
16	A/chicken/Zhejiang/ZS1/2022	H9N2	2022	-	
17	A/duck/Yunnan/YN-9/2016	H5N6	2016	-	
18	A/chicken/Zhejiang/ZJ49/2021	H6N6	2021	-	
19	A/chicken/Germany/N/1949	H10N7	1949	-	

https://doi.org/10.3390/v18080879
